# Effect of gender preference on fertility: cross-sectional study among women of Tharu community from rural area of eastern region of Nepal

**DOI:** 10.1186/1742-4755-11-15

**Published:** 2014-02-14

**Authors:** Pramila Rai, Ishwari Sharma Paudel, Anup Ghimire, Paras Kumar Pokharel, Raju Rijal, Surya Raj Niraula

**Affiliations:** 1 School of Public Health and Community medicine, B. P. Koirala institute of health sciences, Dharan, Sunsari, Nepal; 2 Department of Orthopaedics, B. P. Koirala institute of health sciences, Dharan, Sunsari, Nepal

**Keywords:** Gender preference, Fertility, Contraceptive use, Sex ratio

## Abstract

**Background:**

Son preference is predominant in developing countries especially South Asian countries and its effect is most visible when the fertility is on transition. Nepal is a country in South Asia where the fertility has declined and son is valued highly. This study examines the parent’s gender preference for children and its effect on fertility and reproductive behaviors.

**Methods:**

Study was conducted in Sonapur village development committee of Sunsari district among women of Tharu community of reproductive age (15–49) currently in union and having at least one child. Data was collected by house to house survey. Data was analyzed with IBM SPSS 20 version. Multinomial and binary logistic regression were used to analyze the relationship among variables.

**Results:**

Three hundred women of reproductive age were included in the study. Current average age of the respondents was 31.97 years and mean age at marriage was 18.87 (SD +/-2.615). Child Sex ratio (male: female) of the respondents who didn’t want any more children was 1.41. The birth spacing following male baby was 3.09 years whereas the average birth spacing following female baby was 2.71 years. Age of the respondents and education status of the respondents were also significantly associated with contraceptive practice. Presence of only female children in family significantly increased the desire of other children (AOR = 10.153, 95% CI = 2.357-43.732).

**Conclusion:**

This study finds that the gender preference affects the fertility and reproductive behavior of the respondents and it is necessary to reduce son preference for the health and well being of children and women.

## Background

Sex preference for children has been a salient issue in demographic work in developing countries for a long time. In societies with high fertility regimes, sex preference for children is not a burgeoning issue. When fertility declines a relatively greater impact rises over the course of the fertility transition as parents become increasingly effective in achieving their reproductive goals and even the fertility is inflated because of son preference. Recently the fertility impact of son preference has further intensified by sex-selective abortion, a relatively new practice that is growing rapidly in some Asian countries. Sex selective abortion inflates the sex ratio at birth and lowers fertility [1].

Interpretation of data of Demographic and Health Surveys reveals a desire for a balanced number of daughters and sons or at least one child of each sex [2,3]. Nevertheless, in a wide range of countries, ample preference for sons is found, especially in South Asia and other developing countries in East and Southeast Asia, and North Africa [4-6] showing differential stopping behaviour (DSB) or male-preferring stopping rules. In China [7], Korea [8] or Vietnam [9], and most prominently in India, the role of parental sex preferences in children’s nutrition and medical treatment [10], in child mortality [4], and in reproductive behaviour [5] has been investigated extensively. Women’s contraceptive use and duration of last birth interval are also linked to stopping childbearing after the birth of a son in Nepal [11].

In contrary parents of western countries prefer children of both sexes and are much more likely to have a third and fourth birth if existing children are all of the same sex, indicating a strong preference for children of both sexes. This increased propensity has added around three per cent to the fertility of the cohorts [12]. In Latin America, on the other hand, a slight tendency to prefer girls over boys is observed [3]. In a comprehensive study by Hank and Kohler [13] including a total of seventeen European countries, focusing on the transition from the second to the third child, the authors found no sex preference at all in France and Poland, a preference for a mixed sex composition in Austria, Belgium, Hungary, Italy, Latvia, Slovenia, Spain and Switzerland, and some indication of girl preference in the Czech Republic. Analyzing the data from the United States, Pollard and Morgan [14] also found an evidence of preference for a balance family with at least one son and one daughter.

Three major factors have been identified that manifest such socio-demographic phenomenon. They are economic, socio-cultural and religious utilities. Sons are more likely than daughters to provide family labour on the farm or in family business and support their parents of old age, although there is some recognition that sons are no longer a dependable source of old age support [15-17]. Marriage of son provides additional household help from the daughter in law as well as an economic reward in the form of dowry payments [18]. In the context of patriarchal family system, having one son is imperative for continuation of the family line, and many sons provide additional status to the family. The utility of having sons also arises from the important religious functions that only sons can provide, though both sons and daughters are required to perform certain religious functions [3,19].

Nepal is characterized by strong son preference because of its patrilocal and patrilineal system with certain religious functions that puts emphasis on presence of at least one son in a family. The total fertility rate for women of reproductive age in Nepal fell from around six children per woman to 3.1 in 2006 and 2.6 in 2011 [20], as the fertility is decreasing in Nepal the impact and effect of gender preference for children is more visible in couple’s reproductive behaviour and choices. This combination of positive and negative conditions for fertility decline creates conflicting needs: people desire fewer children, but remain concerned about high infant mortality and the importance of having a son (and subsequently a daughter-in-law) to carry on the family.

## Methods

The study was conducted in rural area of Sunsari district of eastern region of Nepal that spreads from the foothill towards south consisting Plains of Nepal, among women of reproductive age (15–49 years) having at least one living child and currently in union with their partners from Tharu community. They are indigenous group of Terai (plain) region of Nepal and constitute 6.75% of the total population [21]. This group being marginalized economically, socially, educationally and politically and also deprived of various facilities, it was felt necessary to study such feature of this indigenous group. Though there have been studies on this phenomenon, the representation of this indigenous population might be undermined.

The sample size was calculated based on the assumption that sex preference decreases contraceptive use by 24% [11]. Sonapur VDC was selected purposively as this is occupied with high proportion of Tharu people. House to house survey was conducted to collect the data. The questionnaire for the survey included information on socioeconomic and demographic characteristics, their gender preference towards children and subsequent reproductive and fertility behaviours including family size and sex composition desires, contraceptive uses. The study duration was one year and started from July 2012.

In order to compare differences in gender preference, an attempt was also made to quantify these preferences by sex ratio (SR) of last borne child of those respondents who have stopped or intended to stop the childbearing, son preference ratio (SPR) and desire for balance ratios (BR) for respondents with two living children [22]. The son preference ratio was obtained by dividing the percentage respondents with two sons who did not want any more children by the percentage of respondents with two daughters who did not want any more children. The desire-for-balance ratio was computed by dividing the percentage of respondents with two children of the opposite sex who did not want any more children by the percentage of respondents with two children of the same sex who did not want any more children. Respondents with two children who want no more children included in this computation to find son preference and desire-for-balance ratios as national family planning program vigorously advocates a two-child family norm.

We conducted the analysis using Microsoft excel and IBM SPSS 20 version. Univariate analysis was done to calculate the frequencies and percentage. Multinomial and Binary logistic regression were used to see the relation of variables such as sex preferences and socio-demographic variables with fertility intentions and reproductive behaviours. Unadjusted odds ratio (UAOR) was calculated without adjusting with other variables whereas adjusted odds ratio (AOR) was calculated adjusting with other independent variables.

## Results

Total respondents were three hundred married women in union having at least one child. Current average age of the respondents was 31.97 (SD ±8.07), mean duration of marriage was 12.96 (SD ±8.217) and mean age at marriage was 18.87 (SD ±2.615). The respondents had 2 current living children on average.

Relatively higher proportion (22.7%) of the respondents were illiterate in comparison to their counterparts (9.7%). Most of the respondents (60.7%) were housewives. Their socioeconomic status is presented in Table 1.

**Table 1:** Socio-economic characteristics of the respondents (N = 300)

**S. no.**	**Characteristics**	**Frequencies**	**Percentage**
1	Education of the respondent:		
	Illiterate	68	22.7
	Primary	147	49.0
	Secondary and above	85	28.3
2	Occupation of the respondent:		
	Business	15	5
	Agriculture	61	20.3
	Housewife	182	60.7
	Labour	27	9
	Service	5	1.7
	Others	10	3.3
3	Education of the partner:		
	Illiterate	29	9.7
	Primary	135	45
	Secondary and above	136	45.3
4	Economic status of the respondents:		
	Above poverty line	83	27.7
	Below poverty line	217	72.3

The average closed birth spacing was 2.92 years. The average birth spacing following male baby was 3.09 years whereas the average birth spacing following female baby was 2.71 years. Majority of the respondents (88.7%) wanted balanced number and balanced sex composition of the children with one son and one daughter, however, 64.3% of the respondents wanted first child to be a son(Table 2). The sex ratio among those who said they didn’t want any more children was 1.41 (male =138, female = 98). Desire for balance ratio (BR) was 2.17 whereas son preference ratio (SPR) was 1.375.

**Table 2:** Desired fertility behaviour of the respondents (N = 300)

**S. no.**	**Fertility intention and characteristics**	**Frequencies**	**Percentage**
**1**	Desired number and sex composition of children:		
	One son	16	5.3
	One daughter	1	0.3
	Two son	1	0.3
	one son and one daughter	266	88.7
	More son and one daughter	16	5.3
**2**	Sex Preference for first child:		
	Daughter	96	32
	Son	193	64.3
	Whichever	11	3.7
**3**	Decision making for determining no. of children		
	in family:		
	Husband and wife	260	86.7
	Family	14	4.7
	Wife	16	5.3
	Husband	10	3.3

Only 47% of the respondents believed that the sex of the offspring is determined by husband and majority (86.7%) of the respondents revealed that number of offspring is determined by consensus of both husband and wife. We assessed the respondent’s perceived reasons for importance of son and daughter especially categorizing the reasons to be family inheritance; family happiness; religious belief; social security and help in household chores, helpful and understanding nature of gender. Majority of the respondents (79.3%) believed that the female child is important for family happiness whereas 64% of the respondents felt that male child is considered important especially for family inheritance.

Younger aged respondents were significantly more likely to use temporary contraceptives when adjusted with other variables. Having single child (UAOR = 2.988, 95% CI = 1.440-6.198) increased the likelihood of the use of temporary contraceptives. Illiterate and respondents with primary level education were significantly less likely to adopt permanent contraceptives with AOR = 0.173, 95% CI = 0.048-0.621 and AOR = 0.289, 95% CI = 0.097-0.854 respectively compared to respondents having secondary and above education. Likewise respondents having only one child were significantly less likely to adopt permanent contraceptives (AOR = 0.046 95% CI = 0.007-0.294). Having only female children in the family (UAOR = 0.277, 95% CI =0.105-0.732), having last female child (UAOR = 0.375, 95% CI =0.186-0.758) decreased the likelihood of using permanent contraceptives (Table 3).

**Table 3:** Multinomial regression analysis of respondent’s family planning practices (N = 300)

**Current use of contraceptives**	**Independent variables**		**Unadjusted OR**	**CI**	**Adjusted OR**	**CI**
**Temporary contraceptives**	Age	15–24	4.073**	2.072–8.005	3.656*	1.452–9.208
		25–34	3.050**	1.667–5.580	2.837*	1.374–5.860
		35–49^R^				
	Education of the respondents	Illiterate	0.385*	0.189–0.785	0.875	.356–2.149
		Primary	0.557	0.309–1.005	0.677	.350–1.312
		Secondary^R^				
	No. of children	1	2.988*	1.440–6.198	2.148	0.655–7.042
		2	1.668	0.794–3.504	1.330	0.586–3.017
		≥3^R^				
	Sex of last child	Female	0.738	0.449–1.213	0.833	0.377–1.843
		Male^R^				
	Sex composition	Only male	1.478	0.803–2.722	0.519	0.190–1.420
		Only female	0.976	0.541–1.761	0.443	0.170–1.159
		both^R^				
**Permanent contraceptives**	Age	15–24	0.143*	0.032–0.643	0.411	0.066–2.540
		25–34	0.412*	0.184–0.921	0.473	0.185–1.214
		35–49^R^				
	Education of the respondents	Illiterate	0.988	0.372–2.625	0.173*	0.048–0.621
		Primary	1.219	0.520–2.858	0.289*	0.097–0.854
		Secondary^R^				
	No. of children	1	0.104**	0.028–0.384	0.046**	0.007–0.294
		2	0.808	0.384–1.699	0.564	0.237–1.342
		3^R^				
	Sex of last child	Female	0.375*	0.186–0.758	0.416	0.152–1.140
		Male^R^				
	Sex composition	Only male	0.946	0.438–2.045	2.593	0.898–7.489
		Only female	0.277*	0.105–0.732	1.073	0.315–3.655
		Both^R^				

*Significance p < 0.05, **Significance p < 0.001, the reference category is **No use of contraceptives.**

^R^ = Reference.

It was found that model of fit is significant-2 log likelihood = 214.818 χ^2^ (18) = 86.980, p < .001, which indicates this model predicts significantly better, or more accurately.

Current sex composition of having only male children or only female children increased desire of having more children but when adjusted with no. of children and other variables, presence of only female children (AOR = 10.153, 95% CI = 2.357-43.732) in family significantly increased the desire for other children (Table 4).

**Table 4:** Binary logistic regression analysis of respondent’s future fertility intention (N = 300)

**Future plan of child birth**	**Independent variables**		**Unadjusted OR**	**CI**	**Adjusted OR**	**CI**
**Having desire for more children**	Age of the respondents	15–24	10.892**	4.754–24.956	2.650	0.767–9.156
		25–34	4.667**	2.089–10.425	2.185	0.730–6.545
		35–49^R^				
	Education of the respondents	Illiterate	0.145**	0.056–0.373	0.889	0.413–1.915
		Primary	0.353**	0.194–0.642	0.599	0.168–2.140
		Secondary and above^R^				
	Sex composition	Only male	9.545**	3.133–29.082	3.577	0.726–17.634
		Only female	34.650**	11.725–102.401	10.153*	2.357–43.732
		Both male and female^R^				
	Sex of the last child	Female	3.286	1.844–5.853	1.740	0.328–9.215
		Male^R^				
	No. of children	1	13.057**	4.448–38.329	2.472	0.544–11.238
		2	1.317	0.396–4.380	0.806	0.206–3.159
		≥3 ^R^				

*Significance p < 0.05, **Significance p < 0.001.

^R^ = Reference.

The reference category is NOT having desire of more children.

Hosmer and Lemeshow test was used to find out fit of model. It has been found that this model predicts significantly better χ^2^ (8) = 1.858, p >0.05 = 0.985.

## Discussion

This study has shown high sex ratio at last birth and shorter birth spacing following female children. Teen age marriage seemed predominant feature of this group. Plan for next birth was strongly affected by sex composition; women having only female children in family were more likely to plan for another birth compared to others. Age and education of the women, number of current living children were significantly associated with current contraceptive practices.

This high sex ratio at last birth for those who decided to stop child bearing or used permanent contraceptives suggests the childbirth-stopping behaviour was driven by son preference and can be inferred that the son preference behaviour exists in Tharu community. But this might not be totally attributed to son preference as the sex ratio at birth in human populations is essentially a biological constant and for every 1000 males born, there will generally be between 950 and 975 females [23,24] nevertheless son preference and child rearing practices favouring survival of male children could be determinants for this skewed ratio.

Son preference in Nepal has also been demonstrated by other studies too. Higher sex ratio indicating son preference behaviour has been seen in analysis of data from Nepal demographic and health survey 1996, 2001, 2006, 2011 [25,26]. There has been significant fall in the number of girls compared with boys among second-born children where the firstborn was female [26]. It has been found that the average ratio increasing in successive parity in Nepal even reaching 177 boys to 100 girls for third and successive births [27]. The proportion of women who had stopped childbearing whose last child was a boy was much higher than that of such women whose last child was a girl (64% vs. 36%) [11]. Son preference for offspring also ranges according to cultural background even within the country [11,28].

Contraceptive practice being affected by sex composition of the children, number of children, and sex of the last children implies that parents’ reproductive behaviour is more or less influenced by sex preference for children. Other factors like education of the respondents and age of the respondents also have found to exert profound effect on use of contraceptives. It signifies that education, awareness and motivation might be important factors for the practice of contraceptive use when coupled with existing free family planning services that women can afford contraceptives at minimum cost. It has been established that gender preference for children affects reproductive behaviours in other countries as well. Patterns of contraceptive use are concluded to be indicative of a particularly strong preference for sons in Nepal, India, Bangladesh, Egypt, Jordan and Tunisia. In Nepal, women with all sons are five times as likely to use contraception as women with no sons. Women are particularly unlikely to adopt sterilization if they don’t have an adequate number of sons [3]. Study conducted in Sunsari district of Nepal found the preference for son affecting usage of family planning methods [29]. Others study also suggested couple’s desire for number of sons influences fertility differential [30]. The sex of surviving children is strongly correlated with subsequent fertility and contraceptive behaviour and rather than an exclusive son preference, couples strove for one or more sons and at least one surviving daughter [31]. These differentials in contraceptive use, in turn, often affect fertility behaviour as measured by pregnancy rates, the sex ratio at birth, average number of siblings, and sex distribution of children, birth intervals and the duration of postpartum abstinence.

Birth spacing following male child (3.01 vs. 2.71) is longer than that following female child. It implies that the couples want to complete the family by planning another birth as early possible after the birth of daughter whereas after the birth of son, couples take longer time to plan another birth. This finding infers that there is necessity of reducing gender preference for children as evidenced by family planning practices motivated by fertility choices, practices of shorter birth spacing following female child that could harm the health of mother and children. This will also reduce the mental stress of couple raised due to dilemma of small family norm and son preference in the society. If a further drop in fertility is achieved without a commensurate decrease in son preference, the use of sex-selective abortion is likely to increase [11]. Other studies have already shown some of the untoward effect of sex selective abortion which led to excess males [32,33]. Even health care providers believe that illegal sex selective abortion is increasing which may lead to serious abortion complications [26].

## Conclusion

There is intricate association of sex composition and sex of the last child with reproductive behaviours and fertility intention as size of the family, educational level, age of the respondents also play vital role to determine respondents’ reproductive behaviours. That could be effect of decreasing trend of fertility and pressure of small family norm in the society. This study clearly infers that gender preference for children is prevalent in this Tharu community and this affects the fertility intention and reproductive behaviour of the women.

## Competing interest

We declare no competing interest in any area.

## Authors’ contributions

PR is responsible for the conceptualization of the study, designing, data collection, analysis and preparation of manuscript. PKP, ISP, AG, RR and SRN assisted in designing, data analysis, critical review and finalizing the manuscript. All authors read and approved the final manuscript.
